# China Pakistan Economic Corridor Digital Transformation

**DOI:** 10.3389/fpsyg.2022.887848

**Published:** 2022-05-20

**Authors:** Ma Zhong, Majid Ali, Khan Faqir, Salma Begum, Bilal Haider, Khurram Shahzad, Nosheen Nosheen

**Affiliations:** ^1^School of Journalism and New Media, Xi'an Jiaotong University, Xi'an, China; ^2^School of Marxism, Xi'an Jiaotong University, Xi'an, China; ^3^Pakistan Study Centre, University of Peshawar, Peshawar, Pakistan; ^4^Pakistan Study Centre, Islamia College University, Peshawar, Pakistan; ^5^School of International Law, Chinese University of Political Science and Law, Beijing, China; ^6^Department of History, University of Peshawar, Peshawar, Pakistan

**Keywords:** CPEC, digitalization, E-governance, Ajman Digital Government, cloud-computing

## Abstract

The China-Pakistan Economic Corridor (CPEC) vision and mission are to improve the people's living standards of Pakistan and China through bilateral investments, trade, cultural exchanges, and economic activities. To achieve this envisioned dream, Pakistan established the China-Pakistan Economic Corridor Authority (CPECA) to further its completion, but Covid-19 slowed it down. This situation compelled the digitalization of CPEC. This article reviews the best practices and success stories of various digitalization and e-governance programs and, in this light, advises the implementation of the Ajman Digital Governance (ADG) model as a theoretical framework for CPEC digitalization. This article concludes that the Pakistani government needs to transform CPEC digitalization by setting up the CPEC Digitalization and Transformation Center (DTC) at the CPECA office to attract more investors and businesses.

## Introduction

Transformation is smooth only if it starts from inside an organization. Policymakers worldwide are trying to address the immediate economic challenges of Covid-19 by capturing the opportunity for long economic recovery through digitalization. In addition, countries including Pakistan should not wait too long to achieve their digitalization goals. Instead of the unavailability of state-owned infrastructure, Pakistan embraced digitalization in 2020 because of its phenomenal ability to boost the e-commerce of Pakistan (Qureshi, 2021).

In the Covid-haunted world, the physical movement of individuals depends upon country's rules, Covid-SOPs, quarantines, variants, tests, jabs, and boosters. Such an unpredictable situation is scary to ordinary travelers, and especially to investors. Due to CoVID-19, CPEC's physical infrastructure is moving at a slow pace, and is forcing the government of Pakistan to continue its governance and management through technology, as done during the pandemic with the Digital Silk Road (Gyu, 2021).

Our study focuses on the digitalization of CPEC within Pakistan. It discusses the means and ways of adopting digitalization and assesses how policies can be designed to achieve a higher level of digitalization in CPEC. To provide background to this idea, we analyze, assess, and compare E-governance and digitalization policies and initiatives around the globe to highlight the effectiveness and efficiency of digitalization adoption policies. Our study provides a detailed layout of digitalization inspired by different governmentswith the help of an analytical framework, which is based on our argument. It indicates how digitalization policies can be adopted for CPEC-focused initiatives.

Our study aims to pinpoint whether and how digitalization affects CPEC's reputation, as it is considered an economic driver for enhancing Pakistan's economy. Digital transformation in our study refers to all the activities, projects, actions, and procurement that have been undertaken to realize digitalization through DTC and increase CPEC efficiency. Among other factors, improving reputation through the revolution of information can positively impact CPEC and improve its potential to attract more investors and retain consumers. Our study will use the words E-governance, E-Government, and digitalization interchangeably. We have the ultimate aim that the government of Pakistan may adopt it and provide E-Infrastructure to CPEC-related services for various stakeholders. This paper is structured as follows: Section Background: Opportunities and Threats discusses the background of the problem and determines both opportunities and threats to non-digitalized CPEC. Section Rational for Adoption Digitalization Policies endorses the increasing ICT adoption substantial returns to CPEC. Section Effective Digitalization Adoption Policies argues how effective digitalization adoption policies can be outlined, considering how the adoption process works. In Section Comparative Analysis of Digitalization Adoption Policies, a layout of comparative analysis of digitalization adoption policies is discussed. Section Applied Frame of ADG applied ADG to describe, analyze, and compare CPEC Digitalization adoption policies.

### Research Questions and Gaps

Is there any digitalization project that can be used as a role model for CPEC digitalization? What are the hindrances in the way of CPEC digitalization? What literature review suggests the digitalization of multinational projects? What are the assumed benefits of CPEC digitalization?

Teece in 2018 wrote that “Digitalization is a less explored area because of its novelty, however digitalization as a potential booster of organizations and banks performance remained largely unexplored (Forcadell et al., 2020)”. By 2025, digitalization will create six million jobs in logistics and in the electricity industry (World-Economic-Forum, 2021). althoguh further investigation is required to narrow down logistics and electric fields to the Asian context. The machine age may create its own ethics and e-norms that may be taught to future generations. Like in current society, children are taught social studies and ethics in schools. Social media created the hiring of social media account managers, TikTok Live streamers, search engine optimizers, and digital security persons (World-Economic-Forum, 2021). Further exploration is required on whether digitalization is creating jobs or depleting jobs, particularly clerks, admins, artists, and drivers; however countries like Singapore are providing Skill Future Credit to those citizens who are above age 25 and want to develop their industry skills o (Gov-Singapore, 2016). Further research is needed to identify industries, what they need, and what their required skills in the presence of AI are. It is also suggested by some studies that a detailed investigation is required of the risks of digitalization for international businesses. Some studies embarked upon digital interdependence, information security, and the regulatory complexity of digitalization (Luo, 2022).

### Fundamental Problems of Digitalization

Digitilization may control employee's autonomy and their choice of solutions to problems and it may stick employees with developed forms and procedures (Zeshan et al., 2021). In lower level implementation this seems acceptable, but sometimes some problems (like social unrest) are spontaneous and require urgent action, which is not available in the databases. For a complete culture of digital transformation, it will be required to upgrade worker's skills accordingly; this will require further investment in pieces of training (Bernini et al., 2021). Another problem of digitalization is its constant shaping and reshaping status and the threats and risks associated with its changing position financially and psychologically; in the past 50 years it radically reshaped the business associated with it (Brenner, 2018). A grave concern about digitalization is that it may affect jobs, wages, inequality, health, resources, and security (World-Economic-Forum, 2021).

## Background: Opportunities and Threats

**Figure 1:**
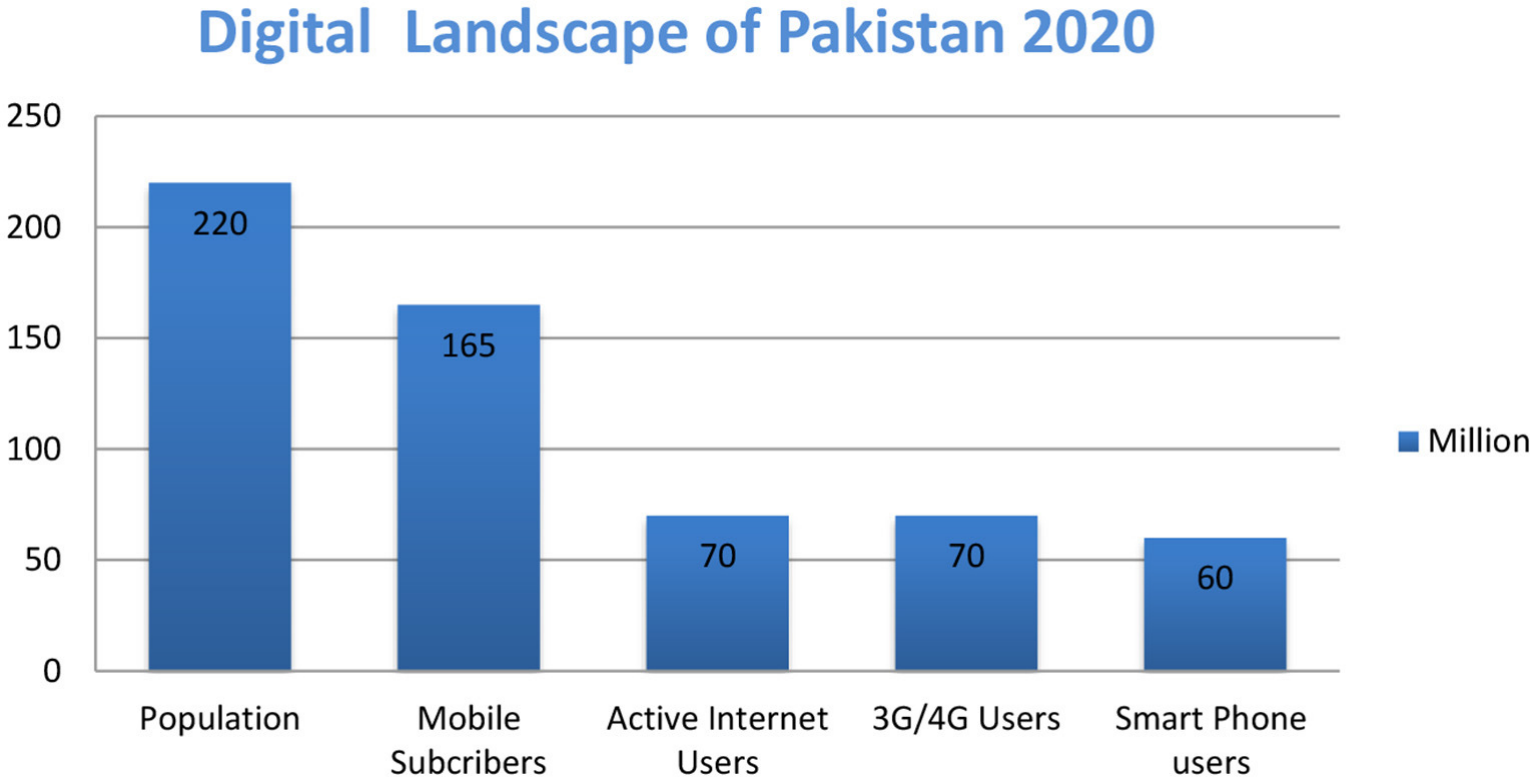
Digital landscape of Pakistan 2020.

The total population of Pakistan is 220 million, of which 165 million are mobile subscribers, 70 million are active internet users, and 60 million are smartphone users. According to the Pakistan Telecommunication Authority, out of 165 million mobile users, 70 million are 3G/4G users, with an increased rate of 0.173 million per month (Figure 1) & (TheNews, 2020). Pakistan is contributing significantly to the technological industry, which can help economic growth and give ground to investments and business in information technology (TheNews, 2020).

**Figure 2:**
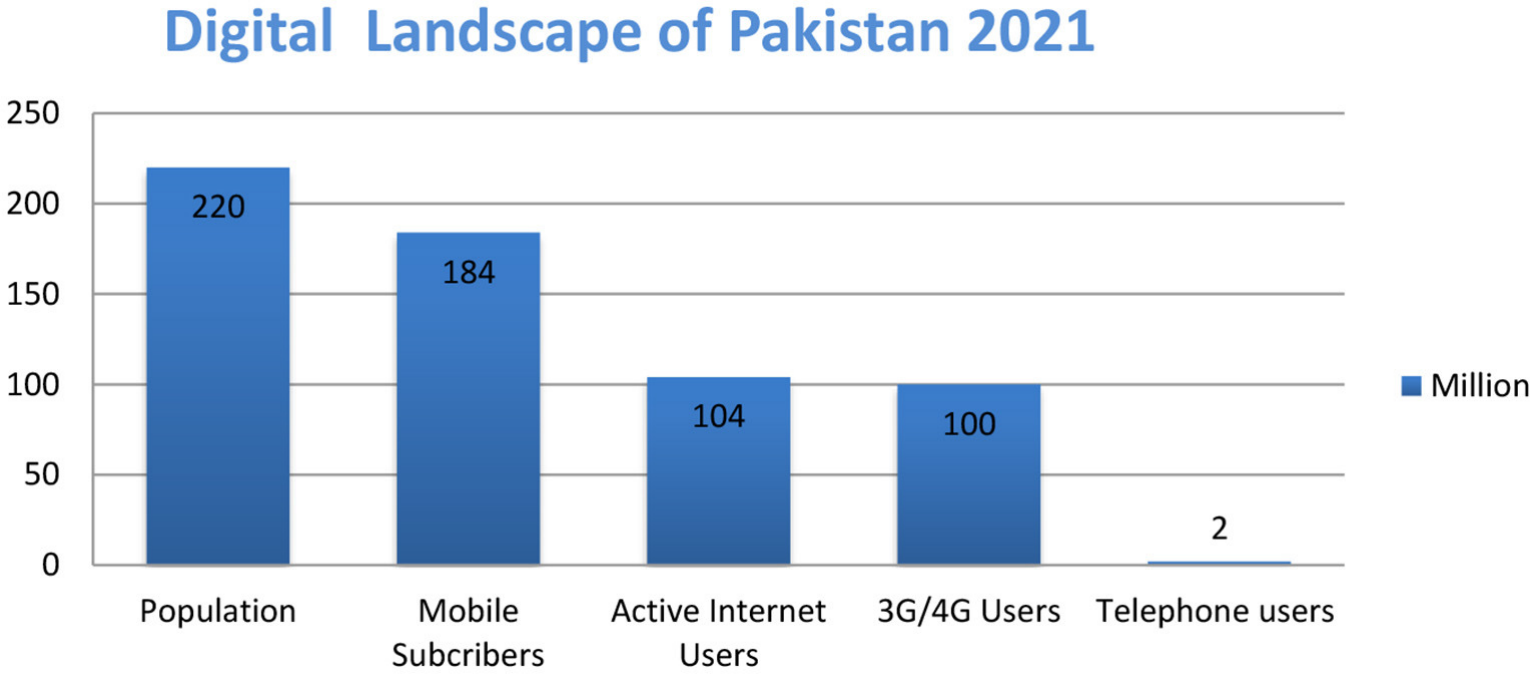
Digital landscape of Pakistan 2021.

Significant growth is observed in all mentioned sectors within 1 year, as cellular subscribers increased from 165 to 184 million, 3G/4G users increased from 70 to 100 million, and broadband internet users increased from 70 to 104 million (see Figures 1, 2). By the end of 2025, 74% of Pakistanis will use smartphones (Ali, 2021).

The Pakistani government has launched some policies for digitalization that reflect the interest and efforts of the Pakistani government. Here, we outline some of the steps Pakistan has taken for digitilization.

### RAAST

Pakistan Instant Payment System is a person-to-person (P2P) instant digital transaction, through mobile phones, conducted among individuals, businesses, and governmental entities instead of going to banks.

Under this initiative, universal access will be provided. All financial industry players, including commercial banks, microfinance banks, and government agencies, will be involved for digital money transfers (Shah et al., 2020).

### Roshan Digital Account

It is a facilitation program of the State Bank of Pakistan (SBP, 2022) in collaboration with commercial banks of Pakistan to provide instant access to opening bank accounts to Non-Resident Pakistanis (NRPs) within 48 h without visiting a bank branch. Under RDA, access to funds transfer, fee payments, E-commerce, investment in the stock market, opening up investments, and the property market will be provided to individuals (Gov-MOFA-PK, 2020). However, this facility is not unavailable to companies and entities (SBP, 2020).

### e-Governance at the Province of Khyber Pakhtunkhwa

Pakistan has experienced e-governance for the first time through Khyber Pakhtunkhwa. The e-Governance, Performance Management, and Reforms Unit (PMRU), established in 2016, ensures effective service delivery, coordination, and oversight of the implementation of sectoral reforms, performance management, and promoting e-governance (Gov-PMRU-PK, 2019).

### Digital Census

Pakistan announced that it will conduct its 7th National Census digitally. A National Census Coordination Center (N3C) will be set up to facilitate the census and Census Support Centers (CSCs) at the tehsil level. These centers will be available 24/7 for smooth implementation. The government plans to purchase 120,000 tablets to implement the 2022 Digital Census. The Federal Minister said, “The census is an important task, so we need to use the latest technology” (Paracha, 2022). The government says the next election (2023) will be based on the results of this digital census (APP, 2022). The reason for this digitized census by the Pakistan Bureau of Statistics (PBS) is the shift from manual to digitalization which took place in 2018 (ProPakistani, 2020). Pakistan successfully implemented E-Governance initiatives of Table-based Price Collection system (TPCS), Consumer Price Index (CPI), Wholesale Price Index (WPI), Sensitive Price Indicator (SPI), Tablet-Based PSLM Survey, Development of Android Based Software for Pakistan Demographic Survey (PDS), Price Analytical Dashboard (CPI,WPI, SPI, RPI), Tablet-Based Software Development for LFS, Revamping of PBS Website, Development of Software for HR, Litigation Management, Inventory Management, and Development of Dashboard for Censuses/Surveys (Gov-PBS, 2021).

There is an urgent need for the digitalization of CPEC for economic growth. To facilitate this, the government of Pakistan established the China-Pakistan Economic Corridor Authority (CPECA). Although its existence and legitimacy are debatable, Pakistan largely manages the overall management of CPEC. The purpose of signing the CPEC is to make Pakistan an economic hub based on technologies such as the Digital Silk Road (DSR). DSR aims to bridge the gap between developed and underdeveloped countries and remove all obstacles in way of development. The objectives of CPEC are also the same: to remove all obstacles in the way of developed and undeveloped areas of Pakistan (Lugt, 2021).

### Statement of Problems

Our study found that CPECA's website does not have sector-specific, geography-focused, and investor-centric information, so would not help an investor decide whether to invest in CPEC, particularly investors not physically present in Pakistan (see for detail http://cpec.gov.pk/). Pakistan is desperately looking for investors. However, Global Finance Magazine did not include it in its 2016 and 2018 foreign investment lists due to its poor governance (OBIOLS, 2018). The government of Pakistan relaxed the visa and citizenship requirements to attract investment (Dawn, 2022). However, this may not be helpful unless it offers monetary and legal incentives. The proposed nine SEZs must have their individualistic benefits to investors. Otherwise, why would an investor from the UK want to go to Pakistan's less lawful areas such as Baluchistan, Khyber Pakhtunkhwa (Merged Areas and settled districts both), and Rural Sindh? Beside that, the government of Pakistan restricted foreigners, including diplomats, from going to these mentioned areas without security involvement (GOV-UK, 2020). Naturally, most investors will opt for Punjab because the UK government declared it safe for citizens. The US government has mentioned the areas of Khyber Pakhtunkhwa and Baluchistan in the “Do not Travel” and “Reconsider Travel” lists (GOV-US, 2021). The US and the UK's reservations show that developed country's citizens may not go directly and would not be interested in such areas or sectors, which is why there is a dire need for digitalization and incentivization of CPEC.

The literature review of CPEC highlights numerous problems and issues faced by Pakistan, but all of them cannot be addressed simultaneously. However, the problems underscored by investors and financial institutions are discussed by this study in detail. Pakistan's entire internet is dependent on landing station cable, which is underwater in Karachi; any disruption can dismantle the whole country's internet. At Gawadar port, an alternative landing station cable is planned, which may minimize the reliance on the already available cable. The Gawadar Cable stretches to Djibouti across the Silk Road from Asia to Africa, underwater over 6,299 Kilometers area. Along with that, 5G technology utilization can remove the risks of internet disruption if it is regularized in line with the prevailing rules of Pakistan. Nonetheless, the whole of Pakistan is not ready for 5G, although at least the CPEC routes, CPEC-SEZs, and concerned offices can be operationalized for interconnecting Pakistan digitally (Rehman, 2020). We can borrow IMPACT 2002+methodology, which is an intergovernmental panel on climate change (Abbas et al., 2021) for CPEC digitalization between China and Pakistan. Or for digitalization, authorities can conduct direct interviews of investors from South Asian countries including China by the multistage sampling techniques used by others (Abbas et al., 2022). One study used a cluster of 80 farmers, and can be replicated on the above mentioned countries to understand the trends investotrs are interested in for digitalization. After developing digitalization, its efficiency can be tested by the ensemble approach (Abbas et al., 2020) used for measuring energy efficiency at Pakistan.

On the other hand, there are some concerns from the international community that the process of CPEC is opaque and unaccountable. These allegations are based on (China Pakistan Economic Corridor Authority Act, 2021) Article 12, which gives complete immunity to its staff from any judicial inquiry. Since the 2019 CPECA's establishment, it did not perform extra-ordinarily (Kiani, 2021). While commenting on the overall situation of CPEC, previous senator Mushahid Hussain mentioned that all opposition parties of Pakistan have concerns over CPECA's establishment, its legality, and staff immunity from legal procedures (Kumar, 2020).

Contrary to that, Pakistan is gradually taking its E-governance initiatives discussed in the above E-Initiatives of Pakistan. However, E-Governance and E-Managed Infrastructure for CPEC does not exist to date, which may hinder attracting businesses and investors. A blogger rightly stated that the government needs to work on policies, rotating around technology and telecoms, develop PPP, offer investor and Business Centric flexible regulations, encourage innovation, devise clear legislation, provide guaranteed privacy to investors, adopt a Citizen-Centric approach, and enhance Digital literacy (Digital-Pakistan, 2021).

Digital illiteracy reduces the opportunities in the new digital market for those who show a low level of ICT adoption, and in the future, they will be unemployed, uneducated, and isolated (Berg and Winden, 2002). It requires adopting a need-based mechanism to increase governmental efficiency and productivity while using technology (Li et al., 2021).

The CPEC digitalization and transformation is totally in line with Government of Pakistan E-Governance initiatives, called the “way forward toward Digital Pakistan,” a strategic framework planned to establish short-term and long-term robust plans for e-government. Under this program, all ministries were expected to adopt digitalization by March 2019. However, the revision did not materialize the whole digitalization package (Gov-IRC-PK, 2020).

There is insufficient focus on CPEC digitalization, which may discourage the project's goals and pose a challenge because countries in the developed world seek sustainable development within digitalization (Dobriansky et al., 2020). In the era of advanced technology, the most applauded approach is the adoption of ICT for efficient results. It is believed that ICT adoption can contribute to sustainability, economic development, and competition (Berg and Winden, 2002). To make CPEC more beneficial in the face of the fourth revolution, there is a dire need for a good revolution of information and e-literacy among its stakeholders. The CPEC digitalization may stimulate E-business, E-governance, and E-management and create new opportunities for stakeholders (Investor and Consumers) to improve Pakistan's Economy.

## Rational for Adoption Digitalization Policies

The manager of thought leadership at A.T. Kearney's Global Business Policy Council, Courtney McCaffrey, was asked at the annual meeting what investors are currently looking for. “They (investors) are focusing on governance and regulatory factors,” she said (OBIOLS, 2018). There are plentiful concerns from investors about Pakistan governance. And this lack of rule of law is confirmed by the World Justice Project Roll of Law Index 2020, which ranks Pakistan 120th out of 128 countries (World-Justice-Project, 2020). For Pakistan, it will be difficult to reform every sector and solve all the problems instantly. Pakistan can take advantage of 62 billion dollars of investment by managing it efficiently and transparently. In the current era, performance and transparency are expected from ICT applications in government, private, business, and public-private partnership (PPP) initiatives, which has a growing impact on the performance of mentioned forums (Li et al., 2021).

### Digital Transformation Center

First of all, the Government of Pakistan should establish a Digitalization and Transformation Center (DTC) for CPEC under new proposed rules of re-organizing the Federal Government proposed by the Institutional Reform Cell (Gov-IRC-PK., 2021). This means the legislation will not stand in the way of the digitalization of the public sector (Plesner and Justesen, 2022). Then it should be linked with the National Information Technology Board (NITB), which is the combination of Pakistan Computer Bureau (PCB) and Electronic Government Directorate (EGD) commissioned by the Ministry of Information Technology and Telecommunication (MoIT) for technical assistance (hard & soft). The purpose of the NITB establishment was to address the operational challenges of all government departments and ministries and implement robust IT technologies to promote an E-Governance culture in the country. As of Feb 22, 2022, NITB has developed 71 websites, deployed E-office in 69 ministries, and provided more than 1,200 IT consultancies to governmental organizations (Gov-NITB-PK, 2022). After finalizing the infrastructure component, the second phase of CPEC is attracting investors and technological transformation, and the installation of industrial, technical, and agricultural technologies for the envisioned development of Pakistan. That is why the President of Pakistan invited the ambassador designated to Korea, Nabeel Munir, and the ambassador to Algeria, Muhammad Tariq, to invite investors from Korea and Algeria to invest in CPEC (Qureshi, 2022).

DTC Digital Governance should consider competing models and the potential challenges of fostering sustainable e-governance. The only benefits of this change are change in strategic decisions, efficient implementation, system platforms, governing culture, and behavior. It has complete IT infrastructure offices and relevant ministries.

Some may ask why Pakistan would not imitate the Chinese SEZ's model instead of taking a hard road with unknown roadblocks? It must be clear that every country has a different political system, and everyone has one's associates and rivals. Therefore, DTC is a concept that will synchronize the needs of Pakistan with the current situation. However, Pakistan needs to build its apps and databases with the help of China and other techno experts. Non-local databases can create a significant gap between CPEC and its investors. Cooperation and collaboration between partners and government officials should not be underestimated(Ghazaleh and Ahmad, 2018).

**Figure 3:**
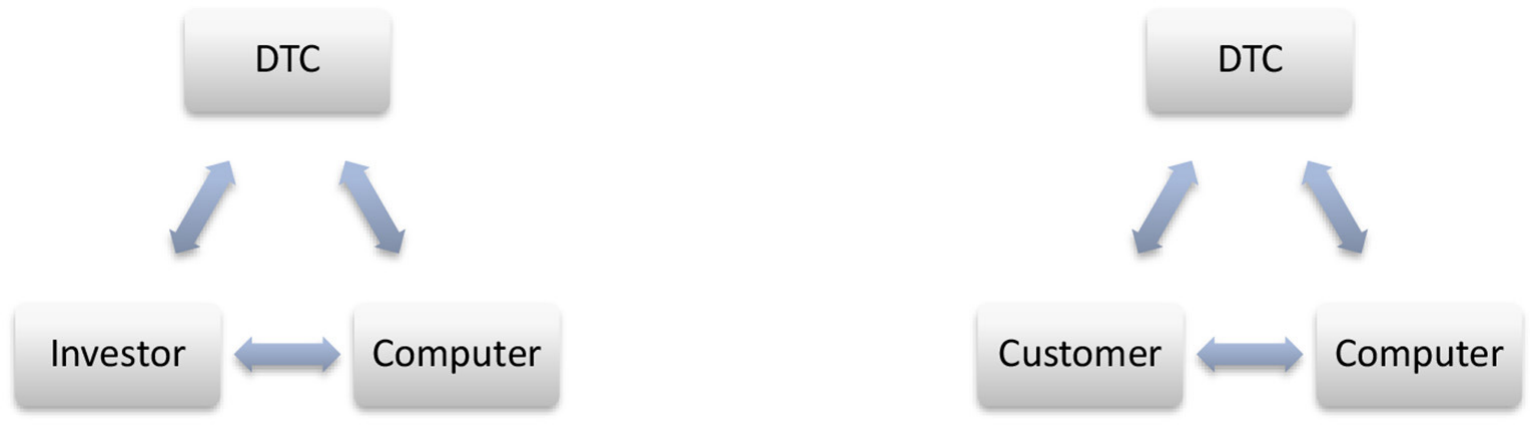
DTC-investors-customers and ICT interconnectivity.

If we apply Forlizzi, it all looks the same. The CPEC will seem like Chinese SEZs, at first glance, or DSRs, as they say, Lady Gaga is the new Madonna (Forlizzi, 2010), but in reality, it is not in the context of Pakistan, because CPEC is the first project of its kind. This is Pakistan's first experience clubbing PPP and the international community in one platform. The cycle illustrated below has some similarities and some differences, which give a picture that both cycles need to combine the interest of each other (Investor & Customer, see Figure 3).

Two years after the deployment of the Belt and Road Initiative (BRI), China launched the Digital Silk Road (DSR) in 2015 to deepen its global trade relationships through E-initiatives. DSR has been compared by one of the tech analysts, Paul Triolo, with the McDonald's business model of franchises everywhere (Wedell, 2020). DSR core components are optical cables and satellite passageways construction and installation for expanding information exchange and collaboration (Lugt, 2021), which is the forerunner of CPEC.

CPEC digitalization may offer modern opportunities to improve information revolution interactivity through the delivery of 24/7 public and private services. It would also involve the consultation of stakeholders in the decision-making process. That is why it is in the economic interest of the Federal Government to find ways for its realization.

#### Defining Goals

DTC can formulate a digital transformation strategy with the involvement of CPEC stakeholders which will have three objectives of change: short-term, medium-term, and long-term. Its sub-goals include the care and satisfaction of investors and consumers. The medium-term goals should be to continue the transformation and achieve 100% digitalization of CPEC for investors, and the long-term goal is to launch e-governance as a whole. This strategy could be achieved by rapidly developing CPEC and DTC targets by developing a roadmap for e-governance through the establishment of an online one-stop service (OSS). As Pakistan establishes a physical OSS, Pakistan needs to establish an online OSS and provide investors with a significant role in governance and decision making.

#### Vision and Mission

The vision statement must say, “Access to high quality, integrated, responsive, and innovative CPEC services anytime from anywhere.” Tony Blair once said that one needs a vision for where one wants to take the country; nevertheless, the hard part is putting the machinery that will make it happen (Volcker, 2014).

## Effective Digitalization Adoption Policies

Here we will discuss how policies for effective digitalization can be designed, considering how the adoption process of digitalization works and how it will start in Pakistan in general and CPEC in particular. This suggests that CPEC-DTC should design policies from the head office down to SEZs to stimulate digitalization for its targeted population. First, CPECA should devise a procedure for establishing DTC. Second, CPECA-DTC should focus on the barriers that keep investors far from investing in CPEC. Third, it should design policies for effective digital transformation for related ministries and government departments. Fourth, it should develop E-Governance, E-Management, and E-services of CPEC.

### Establishment of DTC Policy

**Figure 4:**
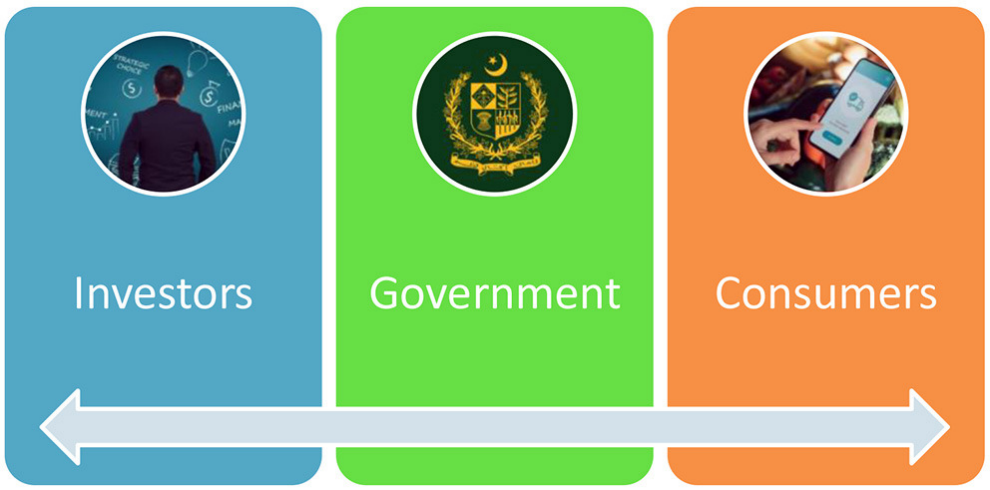
CPEC digitalization and transformation correlation.

DTC will be a governmental organization for CPEC, which will integrate and distribute the information among its stakeholders internally and externally to enhance the business of CPEC and realize Pakistan's envisioned dream of economic and social development (Figure 4). It is an innovative change that can be achieved through solid leadership with an extensive experience in e-governance and e-management. One way to move toward digitalization is to picture the world of the future with touch mode services without paper and pen. Agreements will be sent via email, and bank checks will be confirmed with a simple click or face scan.

#### Legal Options for Digitalization

As the Amir of Ajman established Ajman Digital Governance (ADG) by a decree in 2017, the President of Pakistan may establish Digital Transformation Center (DTC) for CPEC through an ordinance if the elected government cannot pass a law from parliament. The President of Pakistan will issue an ordinance for DTC establishmentbefore the ordinance expiration elected government may present it at the National Assembly, which will become an Act of Parliament. Avoiding internal and external criticism, it is in the best interest of CPEC to establish DTC with the full support of parliament based on the World Bank's devised six principles of rulemaking (World-Bank, 2016). This should be done with two clear objectives: 1) the digital transformation of CPEC and its related authorities, and 2) the electronic management of CPEC. This is because investors are more concerned with its rules and regulations (OBIOLS, 2018). Pakistan needs to clarify all the bottlenecks and mention the rules and regulations about CPEC on its website, which are not currently available (http://cpec.gov.pk/). DTC must keep updating the CPEC website related to investors' interest, with multiple announcements that may keep them focused, otherwise news from inhospitable sources may distract them (Moulton and Leow, 2015).

#### Making Long- and Short-Term Aims

DTC may need to develop a 90-day plan for all three sub-objectives. Then the DTC must test all the E-developed tools, including its links with websites and online e-telepone consultant services. If there is any discrepancy, they need to review and address the issues. It seems a hypothetical layout, but it is not; it is an overhaul plan of CPEC digitalization designed with the help of different e-government projects from Europe down to Asia (consult section comparative e-governments).

#### Digitalization Team

DTC may be headed by the CEO and General Director for implementing its vision as implemented by ADG. The e-Governance of CPEC is a complex idea, which may demand a team of visionary managers who have visions for the future. Who may have political and technical knowledge of resolving issues and have a strategy for interconnectivity with related persons and authorities in and outside the country? Here we find a suggestion from ADG's director of the E-Management team: “ADG Transformation Plan takes on board all governmental agencies and entities that transcends all regulatory and traditional impediments and formed a new team by a higher committee for ADG (Ghazaleh and Ahmad, 2018)”.

In Pakistan, the government department's duty hours start in the morning, except for law enforcement and health departments, and CPECA is not one of them. Through DTC, its staff may be available in shifts for days and nights, facilitating customers and investors through an online help desk. The government of Pakistan already approved establishing a One-Stop Service (OSS) in SEZs to facilitate investors of CPEC, and is helpful in linking with digitalization. It is a one-stop-shop service that provides technical expertise on clearance, licensing, and licensing procedures and requirements required for CPEC investors (CPEC-Info, 2022). This would be how to access OS services that are not physically available in Pakistan. Andhra Pradesh stated that “e-Seva” is a one-stop-shop for both physical visits and e-visits for customers paying utility bills, licenses, certificates, and even bus tickets that can be obtained in person or on the Internet (Auffret, 2010).

### Investors Focused Policies

Pakistan cannot ensure security and safety for the whole of Pakistan at present but can make CPEC more secure with the help of AI, Drones, CCTVs, and other security technology. Investors can get information about their area of interest, geography, established industries, and experiences of those industries from the comments of the users using the online facility on the CPEC website. This can empower an informed decision with the help of DTC, but before that the DTC needs to provide all the information that can be obtained through a suggested route.

Business holders, agents, and companies can check stocks, payment methods, and product chains available through apps and the Internet. They can also place orders online, check duty, and find the nearest way to get stock out of CPEC-Hubs. In a nutshell, the more the information diffuses, the more the investors can affect the business model (Ozsoylev et al., 2014).

CPEC must prefer investor-centric policies and not be generic; otherwise, it may distract the investors. DTC must ensure that the government is not competing with investors and private entities but rather taking them up where they are required, as the private sector may offer to provide free internet to all CPEC routes (Berg and Winden, 2002, p. 269). There should be a CPEC investors Network where the government can share up-to-date information because individual investors' trading behavior and profitability are influenced by their position in the network (Ozsoylev et al., 2014).

### Related Ministries Policies

DTC will implement an overall digitalization plan as a supervisory and executive entity with CPEC and its related 15 ministries. These ministries will provide E-services to CPEC-related initiatives as done by Brazil, where more than 1,600 Government Websites are integrated into a new platform, and more than three hundred government entities are providing e-services (Mari, 2020). Ajman also brought all of the service-providing ministries under ADG (Ghazaleh and Ahmad, 2018), which provides CPEC with a tested path to bring all the related ministries under E-Governance. Nevertheless, before that, E-infrastructure and E-readiness are necessary, for which DTC must equip itself. One study on E-Governance concluded E-readiness in these words “it is a measure often used to gauge how ready is the organization to participate in electronic activities such as e-governance, which is also used to evaluate the quality of ICT infrastructure at the national level or in large-sized organizations (Gupta et al., 2015)“.

**Figure 5:**
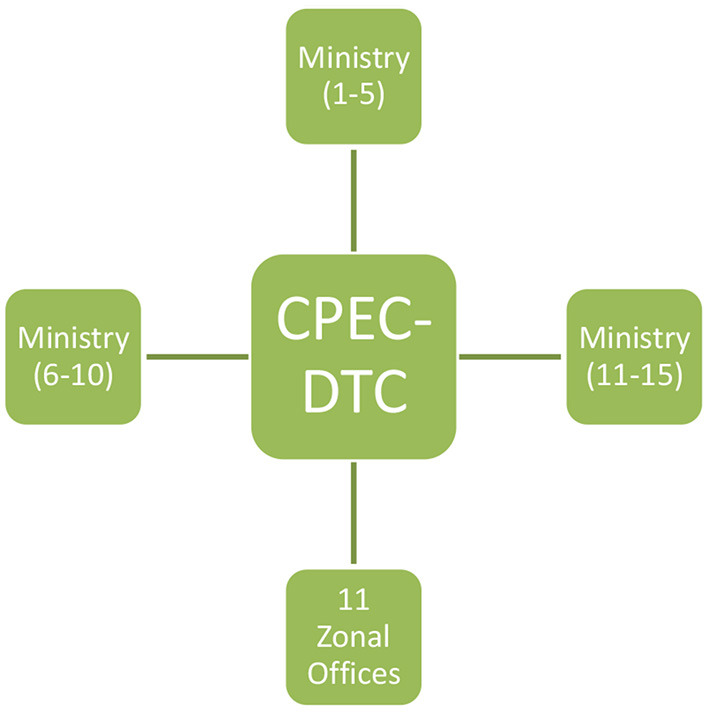
DTC and Ministries interconnectivity.

Still needed is a connectivity service among 15 Ministries with CPECA and DTC through the development of network and applications, along with e-filing DTC, which will digitalize documents (contracts and MOUs) and integrate it with the current workflow. It can be shared, stored, and edited quickly anywhere, with the permission of competent authorities (Figure 5).

DTC will conduct online and physical training for its staff once a month on potential cyber security threats. The DTC Directorate needs to be in the CPECA Secretariat, where it will have a control setup of 11 zonal offices. This is where the individual SEZ will manage the same way the DTC manages the entire CPEC in the center. DTC planners will put the clients' interests above all considerations and manage stakeholders with a coordinated system to support investors and customers.

#### Strategy and Objectives

The transformation strategy of CPECA is divided into three deliverables according to objectives and priorities: 1) CPECA 80% transformation by the end of 2022. 2) Digital transformation of 15 Federal related ministries and have their services shared by 2023, 3) Increase efficiency of CPECA for investors hunting in the first 60 days, and 4) 80% adoption of digital services by the end of 2022.

The central aim of DTC establishment is the transformation of CPEC to digitalization and to improve the decision making of investors and customers by providing them with concrete tools and all facets of CPEC digitalization. It aims to strengthen CPEC governance by re-engineering with technology and E- management, which will require the involvement of all 15 ministries and the CPEC Authority (Figure 5). Recently, Nepal's government initiated such an idea of combining all ministries with one web (see for details, Gupta et al., 2015).

### Policies for E-Governance, E-Management, and E-Services for CPEC

DTC can take advantage of the e-governance structure established for CPEC. The best practices for e-governance structure are discussed at e-governance forums in Amsterdam and Estonia. Here, leaders share their valuable experiences of successful e-government implementation. These forums also showcase specific e-management experiences and experiences of technological change. Perhaps after ADG's success, DTC could arrange a forum to learn and share its experience.

#### Usage of Cloud-Computing

In the next few years, cloud-computing will be one of the top strategic technologies which can improve governmental functions because of its flexibility toward change; ADG has already used it since 2018. The nature of CPEC requires using on-demand access via the internet to applications, servers, tools, databases, and related information. Cloud computing is the best service for on-demand access because of its lower cost, agility, and virtualization (Vennam, 2020).

CPEC growth, its employee's expansion, geographical enlargement, security and backups, storage and warehouses, parking lots, production, air surveillance, Virtual meetings, leakages and breakages, banking and airports, dry ports and seaports, their integration with government websites and application, and all their management could be made possible with the help of Cloud-computing Technology because it has choices for Public Cloud and Private Cloud, based on SaaS (software-as-a-service), PaaS (Platform-as-a-service), IaaS(Infrastructure-as-a-service), and Serverless computing (Vennam, 2020). The public or private cloud selection is left to the stakeholder's choice. ADG's experiment with Cloud computing may provide a layout to DTC's decision, where the director expressed his level of satisfaction in these words: ”The cloud will also facilitate efforts to comply with technical standards. It will ensure users' access to all applications and technical services more quickly and from anywhere and anytime, contributing to increasing staff productivity and reducing the cost of building and maintaining centers (Ghazaleh and Ahmad, 2018)”.

This new system will enhance coordination among government entities and may create a flow of information, directly mitigating the conflicts and impediments between different entities. It will also provide a video call service to fellow authorities, removing ambiguity. Without CPEC digitalization, the web of roads, railway lines, airports, seaports, factories, farms, banks, institutions, law enforcement agencies, warehouses, hotels, recreational centers, investors, customers, and drivers (who will use smartphones, Apps, Websites) cannot achieve the objective of 62$ billion investment. This huge pool of people can only be managed with the help of technical assistance and effective leadership. This can be done at present, because now the population is small, but as the population grows, so will the CPEC stakeholders. CPEC is currently between China and Pakistan, but will soon be linked to Central Asia and the Middle East, by which time the traffic at each outlet will have doubled (Auffret, 2010). Emergency-based digitalization and transformation of CPECA can make it possible to put all the required steps online, including virtual visits. However, legal issues attached to it could be resolved simultaneously.

## Comparative Analysis of Digitalization Adoption Policies

In this section, we show how digitalization may contribute to CPEC development.

**Figure 6:**
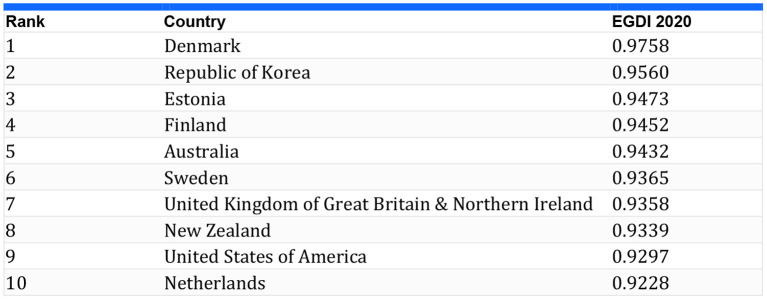
Best E-Governments that were developed with the help of Atlas^1^ and UN-E Governance page^2^.

According to the UN list of e-governments, Pakistan ranks 153rd (Digital-Pakistan, 2021). The reason for this is that Pakistan lacks the infrastructure for digitalization. Denmark, on the other hand, tops the UN-E list due to its digital policies for 2016 and 2020 (Figure 6). Denmark called for interaction between citizens and government agencies, including those who were unwilling to adopt. Another reason Denmark is at the forefront is the digitalization of all its tiers (from central government to municipal), including the private sector and their services. (WorldAtlas, 2021). Some scholars believe E-governance combined with E-administration and E-Services (Bravo, 2021) are the key expected deliverables of E-governance out of CPEC digitalization. One of our study's focuses is on governance and the transparency of CPEC, like with the Korean government. Korea kept the satisfaction of its citizens high; that is why they successfully achieved the purpose of digitalization (WorldAtlas, 2021).

Most of the E-Governance initiatives are influenced by the Estonian model, as is ADG; similarly, it also earned praise in the eyes of scholars who approve of Estonian's e-Governance, calling it “one of the most advanced countries in terms of e-governance.” Under the digitalized system, when ministers have to sign any law, they use e-signatures to reduce the cost of public services for administrative procedures. It reduces public transport usage when citizens come to offices for passport renovation or other official signatures “(Bravo, 2021)“. Although China is 45 on the list of UN E-Governance, most scholars believe that countries like Pakistan can learn from the Chinese e-experience (Bravo, 2021; Ullah et al., 2021).

The modern world belongs to digitalization, which Xi Jinping calls the fourth Revolution (World-Economic-Forum, 2021), where most of the problems will be expected to be solved by technology. However, the most pressing problem for countries like Pakistan is cross-cutting technology governance gaps. Due to its lack of effective regulation, rampant misuse of technology, lack of accountability, privacy and data sharing, Cross-border inconsistencies, and restricted data flows it may face difficulties in the age of technology (World-Economic-Forum, 2021).

### Some Risks of Digitalization in Literature

Controlling the reputation of an organization after its digitalization involves some ris operational, IT-related, and cyber-attack risks (Bernini et al., 2021). There are well-known cyber-crimes, cyber-terrorism, and cyber espionages that pose a threat to national security (Luo, 2022). Through digitalization, massive data will be generated according to a firm's or organization's requirements with its individualistic vulnerability of data privacy, the breakage of that can hinder further digitalization of other organizations (Forcadell et al., 2020). The case of Pakistan is not different; within 1 year (2021-2022) Pakistan saw three instances of data leakage: the National Bank of Pakistan (NBP) on October 30, 2021, the Federal Board of Revenue (FBR) on November 12, 2021, and on November 25, 2021, the hacking of National Database and Registration Authority (NADRA) (Hussain et al., 2022). Digitalization is a double-edged sword; on one hand, it creates opportunities, while on another hand it has complexities, threats, and, vulnerabilities to both businesses and societies (Brenner, 2018; Luo, 2022). i.e. some digitalized companies may create a monopoly and can threaten their adversaries, thereby giving strength to monopolies rather than democracies (Brenner, 2018). The World Economic Forum concluded in one of its reports on digitalization's impact on society that “due to it between two million to two billion jobs will be lost by 2030 (World-Economic-Forum, 2021)”. But the perceived impact of DTC establishment is contrary to WEF calculations because DTC will require hiring tens of hundreds of employees, who will perform assigned duties.

## Applied Frame of ADG

The contents of this study are based on Ajman Digital Government (ADG), which includes Abu Dhabi and Dubai digitization program projects. Ajman established the Central Directorate of ADG to adopt new mechanisms for increasing efficiency in governmental productivity while transforming its public services up to citizens' satisfaction level by adopting digitalization. ADG is a supervisory authority of regulatory and technological affairs that above all permited government authorities that report to the ruler of Ajman. The technological frame of ADG is based on reducing stakeholders' conflicts and improving long-term project planning and implementation. The ADG considered some technological theories like the Technological Acceptance Model (TAM) and Unified Theory of Acceptance and Use of Technology (UTAUT) for its existance.

The UAE Digitalization is referenced as a symbol of accomplishment to other Middle Eastern and North African countries. Pakistan's biggest ever project, CPEC, was inspired by ADG and is an opportunity for its digitalization after Estonia, India, and Brazil.

### Plan and Success of DTC

The successful implementation of DTC's designed plan may focus on investors' problems and their possible solutions, their legal process, the effective E-management of installed businesses, 100% satisfaction of travelers on the route, and other customers. According to its on-service plan, DTC may be in constant coordination through designated 24/7 channels with its required stakeholders because only communication can enhance the project implementation efficiently (Wang et al., 2017).

The Government of India adopted the idea of a 24/7 service partnered with Tata Consultancy Service (TCS) to register companies online (Sharma, 2007); this is another best practice that DTC may adopt for registering new companies and businesses their linkages with banks. The establishment of DTC is not only a technology-driven platform. It is also an organizational transformation project that expects to follow every changing model for meeting CPEC digitalization, focusing on upgrading governmental organizations from an existing environment to a more sophisticated one. The targeted audiences of DTC and ADG are not the same; the latter's result is to give E-services to their citizens as done by other E-Government projects, while the focus of DTC design is investor and customer centric through E-Governance. On one side, it will transform CPEC, while on the other hand, it will attract businesses and investors through its E-Governance and E-management.

The Team of DTC is key to achieving its goals; in the ordinance, the qualification and experience need to be mentioned, not like the CPEC Authority Act 2019, 2020, and 2021 where nothing was said about the qualification of the Chairman and other staff and the selection was left to the Prime Minister's discretionary power. It is better to take guidelines from E-Governance's already implemented projects. Hiring was a significant challenge for the ADG imitative, and they determined the team's qualifications from past experiences. Along with hiring, there is an employee retention problem. ADG adopted the star performers approach closely linked with HR to identify employees worthy of retention. The beauty of E-governance structure is that it can assess all managing cadres' performance by itself, with the help of software and databases. However, CPECA may consult the Job Embeddedness Framework (JEF) for employees retention (Shah et al., 2020). The central database will preserve the input and output taken and given by involved staff, from telephone operators down to the security guards; all will be under software and apps surveillance, which on the one hand will show their performance while on the other hand their effectiveness. It may oversee the implementation of management through scientifically verified means.

### DTC Approach

**Figure 7:**
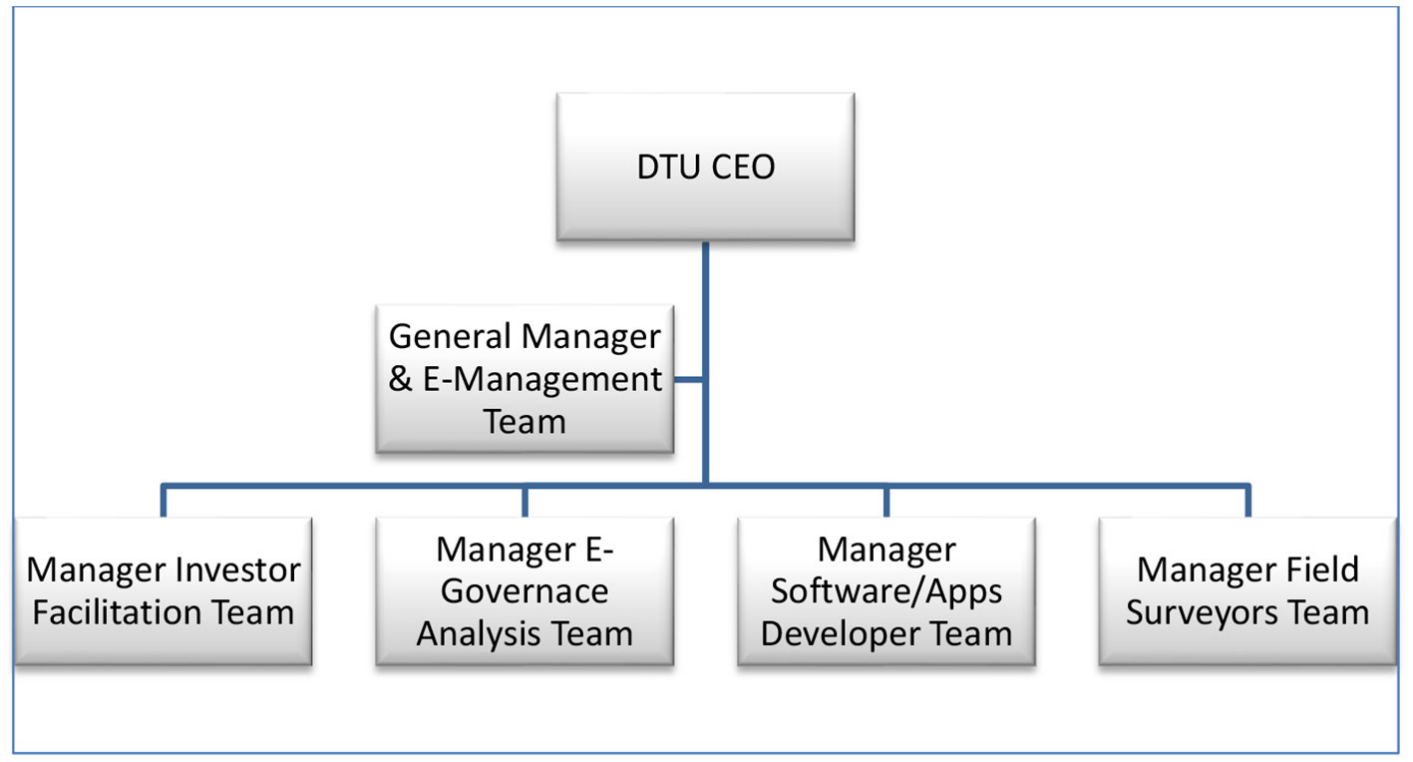
Hierarchical management for DTC.

CEO, Director Generals, and Managers must work together and develop a thirty-day plan with per-day deliverables prioritizing investors' required information and their effective facilitation. The second priority can be the E-Management of investors facilitation teams. The E-Government analysis team, software and app developers, and field surveyors will work under the General Manager and their E-Management team. For the E-management team, developers will develop E-Systems, which can trace every person's growth under the supervision and guidance of the CEO (Figure 7). DTC may follow the structured approach of ADG, whose objectives were to empower employees to accept changes in the desired E-influenced environment. All components of the DTC unit will work in the spirit of Digitalization of CPEC and CPECA Transformation for the future.

DTC will be established in the presence of CPECA, as ADG was established and embedded in the available government infrastructure. It may give birth to complications while procuring some equipment directly from vendors. For the e-government initiatives to succeed, it is required to develop trust among the stakeholders, particularly participants of e-government sister ministries and authorities. Paul A. Volcker believes that we cannot expect trust without excellent and fair public management (Volcker, 2014).

It will be a real challenge for the CEO and their team to achieve the envisioned goals of DTC. To create new jobs and achieve time-bound millstones, it is required to coordinate with governmental authorities and move faster with procurement and hiring. The management may take out budgets, tackle unseen political impediments, manage departmental jealousies, and absorb the pressure of accountability before the court and dare to present their progress to the National Assembly. PM alliance of Atlanta suggests ”A phased approach“ for such a complicated situation, which may successfully go through such adversaries (Stevens, 2009).

### Stakeholders Involvement

In the case of DTC, stakeholders can be investors (national and international), business managers, ministries and and their employees, IT staff, the Chinese and other interested governments, expected consumers, CPECA staff, private sectors, and consultants. The government of Pakistan underestimate the need and importance of stakeholders' involvement in CPEC related decisions; they are crucial for project implementation (Berg and Winden, 2002). The success of digitalization is to meet the expectation of the stakeholders (Mishra and Mishra, 2013). This is the drive of digitalization that minimizes the gap between the old and new setup and adopts such enormous policies, which show the capacity to overcome any resistance with the inclusion of stakeholders' engagement. The changing approach is tough to accept; however, creating an extraordinary sense of urgency for change or creating short-term wins can change the culture (Kotter, 2019).

DTC may form a stakeholder's database from all directly or indirectly involved stakeholders' correspondence with CPECA government departments. DTC stakeholder's network will contribute qualitatively and quantitatively to the needs and expectations of CPEC. The purpose of all efforts is to produce efficient and effective organizations that simultaneously satisfy investors and customers.

DTC can support the investors in the planning and implementation phase of their initiative within CPEC geography and even ask for help from other resourceful persons or entities whose expertise is known to the DTC database. It will keep the doors of learning open for further research and development in technology because unprecedented growth is coming in this sector.

DTC should not underestimate the perpetual revolution and rotation of technological advancements and prepare itself for upcoming new business and management approaches influenced by screen-touched technology. As Harle (2012) stated, everyday growing technical and scientific environment must synchronize techno-human conditions. If DTC management effectively engages with stakeholders, they can achieve the objectives of CPEC.

### Immediate Benefits

- DTC may develop videos for investors and customers and upload them on the CPEC website, reducing the cost of having trained people at Pakistani embassies in countries worldwide to present to the investing community.
- It will provide online helpdesk services (OHS) to investors and Customers to hasten the entire information delivery process while reducing dependency on paperwork, clerical rotations, and passive behavior.
- Instead of rotating in 15 ministries, it will provide multistage interactive information quickly, saving time. That is why Brazil call them Timesaver Centers.
- Placing online orders by customers may enhance transparency and reduce chances of corruption both for a company and the Federal Board of Revenue (FBR) of Pakistan in tax collection.
- DTC case may enhance the trust of investors and customers in Governmental services through strict adherence to the core objective of DTC establishment.
- DTC can develop a Government Electronic Business (GeBiz) system like Singapore did (Sharma, 2007) for online procurement of CPEC-related ministries to bring efficiency and transparency.
- DTC's CPEC-related adopted policies can contribute to economic growth, social rejuvenation, and E-governance, and improve accessibility and trust development.

CPEC, according to its mission, is in search of markets in Central Asia. Now the question is, how will Pakistan reach Central Asian and other East Asian Economies without political Leverage? The answer is digitalization, as the Digital Silk Road enabled full Utilization of the Physical Belt and Road Initiative (Guppy, 2022).

### Importance and Rational of Digitalization Policies

Digitalization in public and private organizations has a positive impact on Human Resource Management (HRM); one study on a French firm reveals that digitalized HRM empowers the organization (Zeshan et al., 2021). Digitalization is a process of improvement in an organization's properties through the combination of ICT and connectivity (Bernini et al., 2021). To predict the future of industries, firms, and businesses ahead for 2 years or 3 years, big data may help create opportunities and risks (Brenner, 2018). Such an estimated prediction may give birth to another discipline, like the machine age (World-Economic-Forum, 2021). In Pakistan, there is a grave concern in provinces and regions about the uneven behavior of the Central government regarding CPEC; the establishment of DTC can add value to society from this digital transformation. In realizing CPEC transformation the vision of digital Pakistan can help the project and bring economic and social benefits if the transformation journey is focused on helping clients and investors. As is mentioned in a Forbes articles, “Business success depends on having a clear understanding of profitability across service lines, customers, and regions (David, 2020)”. Nothing can stop CPEC from achieving its envisioned goals if it qualifies the Forbes demarcated lines of doing business.There are two apparent demands from ICT's adaptation to improve efficiency and to bring transparency by the usage of computer technology, telecommunications, electronics, and media (Berg and Winden, 2002). According to the UN-E government Development Index of 2018, Pakistan ranked 148 out of 193 countries. It ranked 153rd in 2019 due to a lack of digital infrastructure, unaffordability, low digital skills, data insecurity and privacy, cyber threats, trust deficit, and unavailability of IT service design (Digital-Pakistan, 2021).

International tourism to Pakistan decreased in 2014 and recovered in 2018 with around 17, 823 tourists in Pakistan on tour Visas; the rise was attributed to the availability of online visas (GulfNews, 2019). Before 2017, Pakistan was 135th out of 136 countries due to visa requirements; out of 68 commenters on this article, 10 expect the rise in tourism in the future in Pakistan due to CPEC (Ahmad, 2017).

## Conclusion

CPEC is the appropriate spatial-administrative level to pursue digitalization policies and their execution. Digitalization policies can also be designed by parliament with the help of stakeholders. A study of different E-Governance best practices showed the high E-governance and Digitalization results from sufficient material, social, cognitive, and individualistic resources. The secret of the top ten successful E-governance projects is their Citizen-Centric approach and the commitment of high-level government officials.

If DTC adopts the investor- and customer-centric approach, CPEC could be one of the success stories of E-Governance and social transformation both economically and technologically, providing services to its citizens satisfactorily. To impart transparency and sustainability in CPEC, the Government of Pakistan should consider the idea of digitalization of CPEC through a proposed solution; this model is also recommended for Belt and Road Partner countries, those that have a democratic set up with a multi-party system, where transparency and accountability is a grave concern. It is an opportunity for low E-governance systems to enhance their governance for BRI because it has much potential for PPP and government support.

The digitalization of CPEC through the establishment of the CPEC digitalization and Transformation Center (DTC) would yield benefits in the present by attracting investors. In the future, benefits would be gained by satisfying customers and investors by developing software and apps for rail-systems and its E-management, road surveillance and its E-Management, speeding up cross border clearance of custom requirements, farms management, Account opening for foreign investors, Hotels linking with apps, translation of apps, installation of scanning machines and its linkage with Federal database of FBR, E-Tax submission, Monitoring food processes, farming and enabling it to be environmentally friendly, and availability of both countries' Chamber of Commerce with the help of AI for any issue (Guppy, 2022).

## Author Contributions

MZ, MA, and KF contributed to the conception and design of the study. SB and BH organized the database. NN performed the statistical analysis. KS wrote the first draft of the manuscript. MA, MZ, SB, and HB wrote sections of the manuscript. All authors contributed to manuscript revision, read, and approved the submitted version.

## Conflict of Interest

The authors declare that the research was conducted in the absence of any commercial or financial relationships that could be construed as a potential conflict of interest.

## Publisher's Note

All claims expressed in this article are solely those of the authors and do not necessarily represent those of their affiliated organizations, or those of the publisher, the editors and the reviewers. Any product that may be evaluated in this article, or claim that may be made by its manufacturer, is not guaranteed or endorsed by the publisher.

## Footnotes

^1^ https://www.worldatlas.com/articles/countries-with-highly-developed-e-governments.html

^2^ https://publicadministration.un.org/egovkb/en-us/data-center
